# Metformin inhibits cervical cancer cell proliferation by modulating PI3K/Akt-induced major histocompatibility complex class I-related chain A gene expression

**DOI:** 10.1186/s13046-020-01627-6

**Published:** 2020-07-06

**Authors:** Chenglai Xia, Chang Liu, Zhihong He, Yantao Cai, Jinman Chen

**Affiliations:** 1 South Medical University Affiliated Maternal & Child Health Hospital of Foshan, Foshan, 528000 China; 2 School of Pharmaceutical Sciences, Southern Medical University, Guangzhou, 510150 China; 3 Foshan Women and Child hospital, Foshan, 528000 China

**Keywords:** Metformin, Histocompatibility complex class I-related chain a, Cervical cancer, PI3K, Cell proliferation

## Abstract

**Background:**

Recent studies have shown that the classic hypoglycemic drug metformin inhibits tumor growth; however, the underlying mechanism remains unclear. We previously showed that metformin disrupts the sponge effect of long non-coding RNA MALAT1/miR-142-3p to inhibit cervical cancer cell proliferation. In this study, we interrogated the ability of metformin to modulate the anti-tumor immune response in cervical cancer.

**Methods:**

The cell counting kit-8 assay was used to detect the viability of cervical cancer cells. Flow cytometry assays were performed to measure cell apoptosis and cell cycle. Lactate dehydrogenase (LDH) cytotoxicity assay was used to detect NK Cell Cytotoxicity. Relative protein levels were determined by immunoblotting and relative gene levels were determined by quantitative real-time PCR. Tumor Xenograft Modeling was used to evaluate the effect of metformin in vivo*.*

**Results:**

Metformin inhibited cervical cancer cell proliferation, cervical cancer xenograft growth, expression of PCNA, p-PI3K and p-Akt. Moreover metformin induced cervical cancer cell apoptosis and caused cancer cell cycle arrest. In addition, metformin upregulated the expression of DDR-1 and p53 in human cervical cancer cells. Furthermore, metformin also regulated the mRNA and protein expression of MICA and HSP70 on the surface of human cervical cancer cells via the PI3K/Akt pathway, enhancing NK cell cytotoxicity.

**Conclusions:**

In conclusion, our results suggest that metformin may be used as immunopotentiator to inhibit cervical cancer progression and may be considered a viable candidate for combination therapy with immunotherapy.

## Background

According to the tumor data reported in 2019, nearly 529,000 newly diagnosed cases of cervical cancer are reported annually and the mortality of cervical cancer ranks the second in 20- to 39-year-old female cancer patients, with approximately nine cancer patients die of cervical cancer each week [1]. Cervical cancer is generally treated by surgery, chemotherapy, or radiotherapy alone or in combination [2]. However, the survival rate of cervical cancer patients remains relatively low. Although the 2-, 4-, and 9-valent human papillomavirus (HPV) vaccines have been shown to prevent cervical cancer, these do not have protective effects on all individuals [3, 4]. Hence, the development of novel treatments to inhibit tumor invasion and migration and improve the survival rate and quality of life of cervical cancer patients is particularly important.

Metformin is currently the first-line oral drug for the treatment of type 2 diabetes; it is inexpensive and imparts a significant therapeutic effect. Studies have shown that diabetic patients with metformin treatment have a lower incidence of cancer. The antitumor effect of metformin is closely related to mechanistic target of rapamycin complex 1 (mTORC1), a key protein of the PI3K/Akt/mTOR pathway [5–8]. Studies have shown that phosphatidylinositol 3-kinase (PI3K), epidermal growth factor receptor, extracellular signal-regulated kinases, anti-apoptotic B-cell lymphoma 2 (Bcl-2) pathways and proteins play an important role in the development of tumors, particularly solid tumors such as cervical cancer [9, 10]. These signaling pathways and proteins form the regulatory network of cervical cancer and are ideal targets for the development of antitumor drugs. The PI3K/serine-threonine kinase/rapamycin pathway has recently been shown to be closely related to the growth and proliferation of solid tumor cells, and serine/threonine kinases play a central role in the pathway. PI3K inhibitors inhibit the expression of downstream serine/threonine kinases. In addition, serine/threonine kinase inhibitors suppress tumor cell proliferation [11, 12].

Natural killer (NK) cells are important immune cells in the body that are involved in antitumor, antiviral infection, and immunomodulation processes. Under specific conditions, NK cells can identify target cells and activate immune cells. NK cells impart broad spectrum antitumor effects and do not show specificity or major histocompatibility complex (MHC) restriction. NK cells have two anticancer effects. First, NK cells can directly kill tumor cells by releasing perforin and granzymes or by death receptor engagement. Second, NK cells can secrete cytokines and chemokines to activate T cells and exert a killing effect [13]. Studies have shown that valproic acid (VPA) upregulates the expression of human histocompatibility complex class I-related chain A (*MICA*) and histocompatibility complex class I-related chain B (*MICB*) by activating PI3K/Akt signaling [14, 15]. The NK group 2D (NKG2D) receptor is an activated receptor for MICA on NK cells that recognizes MHC class I molecules and plays an important role in innate immunity. When MICA on the surface of tumor cells is activated, NK cells can recognize the MICA and initiate the antitumor immune response [16, 17]. In vitro studies have shown that peripheral blood NK cells (PBNK) are able to kill HPV-infected cell lines [18]. However, NK cells are often dysfunctional and low in number in cervical cancer patients and thereby unable to mount efficient cytotoxicity against tumors [19].

Our previous study has shown that metformin disrupts the sponge effect of long non-coding RNA MALAT1/miR-142-3p to activate the expression of the downstream antitumor protein high-mobility group A protein 2 (HMGA2) and exert an anti-cervical cancer effect [20]. We also found that a combination treatment with metformin and nelfinavir for 12 h significantly upregulated MICA protein expression in SiHa and HeLa cells [21]. However, the molecular mechanism by which metformin upregulates MICA remains unclear. This study aimed to elucidate the molecular mechanisms by which metformin activates antitumor immunity.

## Materials and methods

### Cells, reagents, and antibodies

In this study, the human cervical cancer cell lines, SiHa cells and HeLa cells, and the NK-92 cells were from a research laboratory of Foshan Maternity and Child Health Care Hospital, Guangdong Province, China. The SiHa and HeLa cells were cultured in Dulbecco’s modified eagle medium (DMEM) containing 10% fetal bovine serum (Invitrogen, Carlsbad, CA, USA) and 1% streptomycin (Sigma-Aldrich, St. Louis, MO, USA). The NK-92 cells were cultured in alpha-minimum essential medium (ɑ-MEM) containing 100 U/mL interleukin (IL)-2. Dimethyl sulfoxide (DMSO), LY294002, and metformin hydrochloride were purchased from Sigma-Aldrich. Anti-MICA, anti-MICB, anti-ULBP-2/5/6 Phycoerythrin, anti-ULBP-1 Alexa Fluor 488, anti-ULBP-3 Allophycocyanin, anti-DDR-1, anti-HSF-1, anti-PI3K (110ɑ), anti-phospho-PI3Kp55 (Tyr199), anti-Akt, anti-phospho-Akt (ser473), anti- proliferating cell nuclear antigen (PCNA), anti-p53 primary antibodies, and their corresponding secondary antibodies were purchased from Cell Signaling Technology (Beverly, MA, USA).

### Analysis of cell proliferation

According to the methods described in a previous study [22], cells were trypsinized and seeded in 96-well plates (1 × 10^4^ cells per well). After the cells were attached, metformin was added at various concentrations (0–400 μM) for 48 h. To analyze cell viability, the cell proliferation assay, cell counting kit-8 (CCK-8), was used to measure the absorbance of the samples using a microplate reader and at a wavelength of 450 nm.

### Cell apoptosis assays

According to the methods described in a previous study [8], Cervical cancer cell lines (HeLa and SiHa) were seeded at a density of 2 × 10^5^ cells/well in a 6-well culture plate. The cells were washed twice with cold PBS, and then resuspended in 1× binding buffer. One hundred μl of the cell suspension (1 × 10^5^ cells) was transferred to a 5 ml culture tube and mixed with 5 μl of FITC anti-Annexin V and 5 μl PI. The mixture was gently vortexed and incubated for 15 min at RT (25 °C) in the dark. Then, 400 μl of 1× binding buffer was added into each tube. The samples were analyzed by flow cytometry within 1 h. The green fluorescence of Annexin V-FITC was measured at 530 nm, and the red fluorescence of PI was measured at 585 nm. The results were analyzed with FlowJo software.

### Cell cycle assays

Cells were harvested in PBS and fixed by addition of ice-cold 70% ethanol with a Pasteur pipette during vortexing, as previously described [8]. Then, the cells were centrifuged at approximately 2000 rpm for 5 min and washed twice with PBS. Finally, the cell were stained by PI staining solution (Invitrogen) and analyzed by flow cytometry collecting 25,000 events per sample. The results were analyzed with FlowJo software.

### Nuclear protein extraction

According to the methods described in a previous study [23], cells were washed three times with pre-cooled phosphate buffered saline (PBS) and subsequently scraped off with a cell scraper and collected in a 1.5 mL centrifuge tube. The cells were centrifuged at 1000 rcf, 4 °C, for 3 min. A protease inhibitor, cell lysis buffer, and cell membrane rupture solution were added into the pellet to lyse the cells on ice for 1 h, followed by 1000 rcf centrifugation at 4 °C for 20 min. The supernatant was discarded, and the pellet was then lysed with nuclear extraction lysis buffer on ice for 1 h, with vortexing every 5 h for complete lysis, which was followed by 12,000 rcf centrifugation at 4 °C for 15 min to collect the supernatant (i.e., nuclear protein extract).

### Lactate dehydrogenase (LDH) cytotoxicity assay

As described in the methods of a previous study [21, 23], the human cervical cancer HeLa or SiHa cells were seeded as target cells in 96-well plates (2 × 10^4^ cells per well) in a total volume of 100 μL per well. The cells were then divided into five different groups as follows: drug treatment; effector cells (NK-92) with spontaneous LDH efflux; target cells with spontaneous LDH efflux; target cells with maximum LDH efflux; and volume correction (no cells) groups. Approximately 10 μL of lysis buffer (10×) was added to each well of cells and then incubated in a 37 °C incubator with 5% CO_2_ atmosphere. After 250×*g* centrifugation for 3 min, 50 μL of a reaction solution were added to each well of the 96-well plates, followed by 50 μL stopping solution, with gentle mixing, and then the absorbance at wavelengths of 490 nm and 680 nm were read. The cytotoxicity of different target ratios (%) was calculated using the following formula: Cytotoxicity (%) = (Experimental group − Effector cells with spontaneous LDH efflux group − Target cells with maximum LDH efflux group)/(Target cells with maximum LDH efflux group − Target cells with spontaneous LDH efflux group) × 100.

### RNA extraction and quantitative real-time PCR

According to the methods described in a previous study [20], After transfection for 48 h, total RNA was isolated from SiHa and HeLa using TRIzol reagent (TAKARA, Dalian, China) according to the manufacturer’s instructions. For mRNA quantification, RNA was reverse transcribed into cDNA using the PrimeScript™ RTreagent Kit (Takara, Japan) and then quantified on the CFX96 touch q-PCR system (BIO-RAD, USA) with SYBR Premix Ex Taq Kit (Takara, Japan) according to the manufacturer’s protocols. In this study, GAPDH was used as an internal control for determining the levels of HSP70 and MICA. The primers used for quantitative real-time polymerase chain reaction (qRT-PCR) are listed in Table 1. Reactions were performed using a SYBR Green kit (TAKARA, Dalian, China), according to the manufacturer’s instructions. Each 20-μl reaction mixture included 2 μl of cDNA, 10 μl of SYBR Green Mix, 0.4 μl of forward primer, 0.4 μl of reverse primer, 0.4 μl of RoxReference Dye, and 6.8 μl of RNase-free water. Then, the PCR reactions were performed in the CFX96 touch q-PCR system (BIO-RAD, USA) under the following conditions: 95 °C for 30 s, followed by 40 cycles at 95 °C for 5 s, 60 °C for 30 s, 95 °C for 15 s, and 60°Cfor 60 s. Relative gene expression was determined by using the ΔΔC_t_ method. Significance was defined according to *P* values from the two-tailed t-test. All of the reactions were performed in triplicate.

**Table 1:**
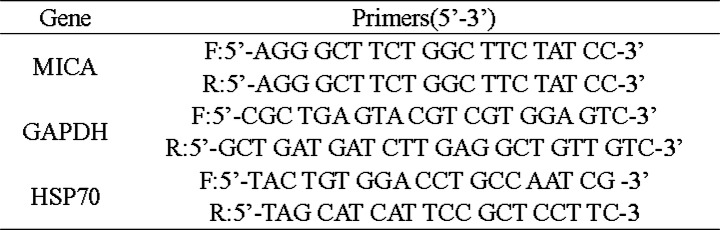
Oligonucleotide primer sequences for qRT-PCR

### Western blotting

Western blotting was performed as previously described [24]. Briefly, the cells were harvested and lysed with RIPA lysis buffer, and the concentration of the collected proteins was determined. Then, 100 μg of the extracted protein was separated in 10, 8%, or 5% SDS-PAGE gel based on the molecular weight of the target protein. The separated protein gel with a pre-stained protein marker was transferred onto a PVDF membrane. Subsequently, the membrane was blocked in a 5% skim milk solution at room temperature for 2 h, followed by incubating with the corresponding primary and secondary antibodies and washing with Tris-buffered saline, 0.1% Tween 20 (TBST) in between. The PVDF membrane was developed using an enhanced chemiluminescence solution (Pierce) and subsequently photographed in a Bio-Rad gel imaging system. The exposure time was adjusted according to the protein bands and background. After selecting the clear protein bands in the image, the gray value of each protein band was analyzed by software and statistical analysis was conducted.

### Tumor Xenograft modeling and in vivo experiments

BALB/c nude mice of 4 weeks old (weighing approximately 15–17 g) were purchased from Guangdong Medical Laboratory Animal Center (Guangdong Province, China). All mice were housed and bred in a specific-pathogen-free (SPF) grade animal facility, with 22–25 °C temperature, 40–60% humidity, and 12 h/12 h light/dark cycle. To generate tumor xenograft, 20 mice were used. The skin of the left forelimb near the armpit was disinfected and 0.1 mL SiHa cells suspended in serum-free medium (containing approximately 5 × 10^6^ cells) were injected. After inoculation of the cervical cancer cells, the nude mice were continuously housed under the same conditions. Once the subcutaneous nodules grown to a rice grain size (required approximately a week), the subcutaneous xenograft model of cervical cancer in nude mice was successfully constructed. The subcutaneous tumor size in each nude mouse was measured using a digital vernier caliper. Once the tumor diameter reached approximately 0.3–0.5 cm, the nude mice were numbered, randomly divided into four groups (with five mice per group), namely, control, model, 50 mg/kg/d metformin, and 250 mg/kg/d metformin groups. Metformin was given by gavage. All nude mice were closely monitored for tumor growth, skin condition, and behavior daily and any tumor ulceration or irritation was noted. The longest (A) and the shortest (B) diameters of the subcutaneous tumors were measured with a digital vernier caliper before each metformin administration to calculate the tumor volume (V) using the following formula: V = 0.5 × A × B^2^. In addition, all nude mice were weighed daily, and their daily food intake was also measured. After the completion of the 23-day metformin administration, all nude mice were sacrificed and placed on ice, their skin was immediately cut open, and the subcutaneous tumor xenografts were collected. After weighing each tumor xenograft on a digital scale, one part of the tumor tissue was dissected and frozen in liquid nitrogen for western blotting. All experimental procedures were approved by the Institutional Animal Care and Use Committee of South Medical University.

### Statistical analysis

The SPSS16.0 software (IBM SPSS, Chicago, IL, USA) was used for data analysis in this study. An independent T test was used to compare the results between the two groups. Multivariate ANOVA was used to compare differences between multiple groups, followed by multiple corrections using the Bonferroni’s test. *P* < 0.05 was considered statistically significant.

## Results

### Metformin inhibits the proliferation of cervical Cancer cells

**Fig. 1:**
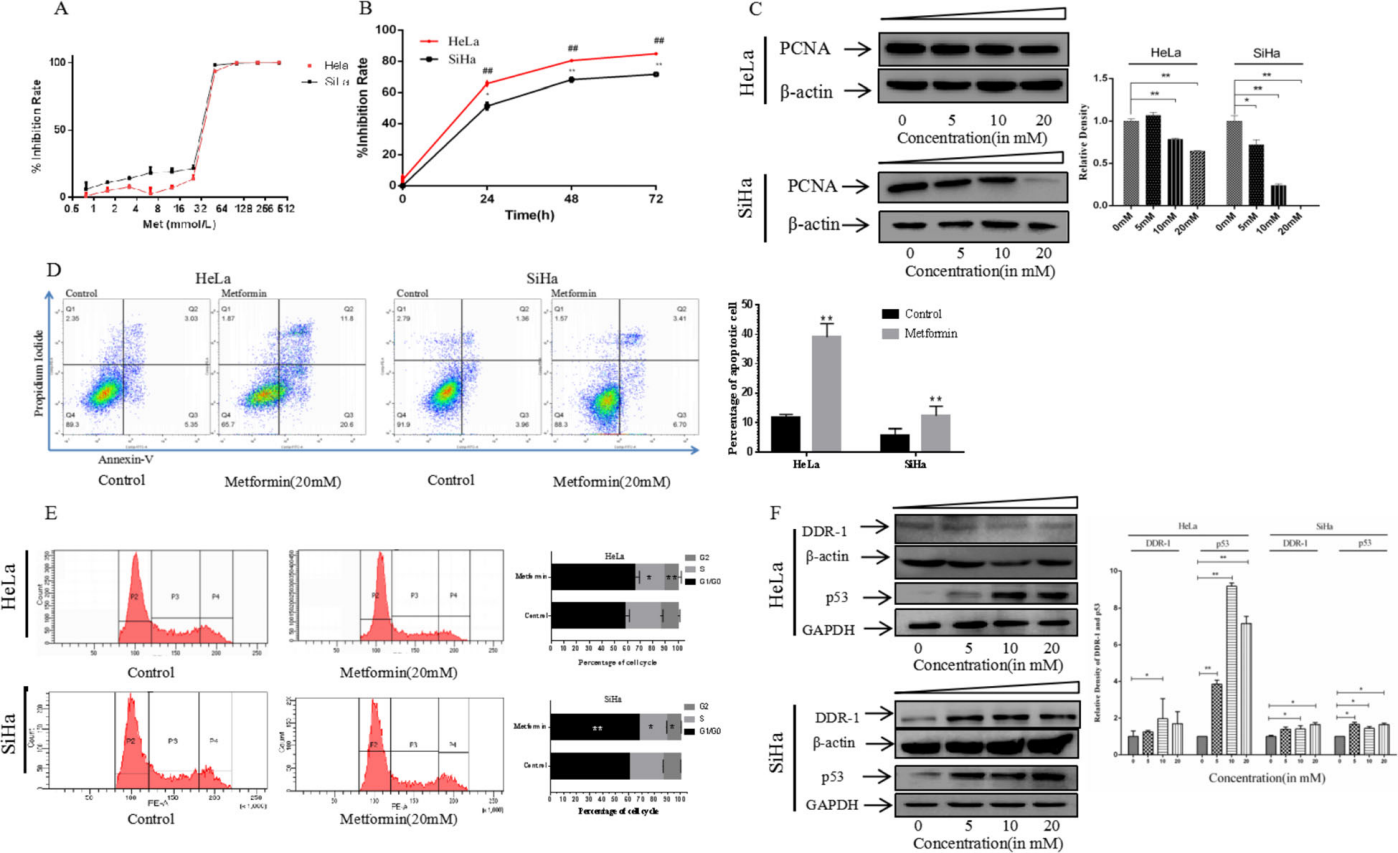
Metformin inhibits cervical cancer cell proliferation. **a** Proliferation of the SiHa and HeLa cell lines with 48 h metformin treatment measured by CCK-8; **b** Proliferation of the SiHa and HeLa cell lines incubated with metformin at the indicated concentrations for 24, 48, and 72 h and measured by CCK-8; **c** Representative western blots and a bar chart showing PCNA expression in SiHa and HeLa cells treated with different concentrations of metformin for 48 h. **d** Apoptosis assays of the SiHa and HeLa cell lines incubated with 20 mM metformin for 48 h. **e** Cell cycle assays of the SiHa and HeLa cell lines incubated with 20 mM metformin for 48 h. **f** DDR-1 and P53 expression in SiHa and HeLa cells after 48 h treatment with metformin of different concentrations as measured by western blotting. Data are presented as mean ± standard deviation, *n* = 3. *P < 0.05, and **P < 0.01, comparison with the 0 μM metformin treatment group

Similar to other tumors types, cervical cancer cells have infinite proliferative properties [25]. Inhibition of tumor cell proliferation has been one of the strategies for developing chemotherapy drugs. Here, metformin was used to treat cervical cancer cells for 72 h, and CCK-8 was used to evaluate the effect of metformin on cervical cancer cell proliferation. The IC50 value of metformin was 25.13 ± 0.99 mM and 19.43 ± 1.41 mM in HeLa cells and SiHa cells, respectively (Fig. 1a). Metformin also inhibited cervical cancer cell proliferation in a time-dependent manner, when cervical cancer lines were treated with metformin for 24 h, 48 h and 72 h (Fig. 1b). PCNA is a molecular marker of cell proliferation and is commonly used in evaluating cell growth status (Branca et al., 2007). Detection of PCNA in the nuclear protein extracts of SiHa and HeLa cells by western blot indicated a decrease in PCNA following treatment with metformin at 5 mM, 10 mM, and 20 mM (Fig. 1c).

Promoting cancer cells apoptosis is a characteristics of chemotherapy drugs. In our present study, we used flow cytometry to detect the effect of metformin on inducing cervical cancer apoptosis. As shown in Fig. 1d, the apoptosis ratio of HeLa cells increased from 11.61 ± 0.47% to 39.04 ± 1.88% and the apoptosis ratio of SiHa cells increased from 5.69 ± 1.02% to 12.31 ± 1.63% when these cells were treated with 20 mM metform for 48 h. Inducing cancer cell cycle arrest is another characteristics of chemotherapy drugs. In our present study, we also used flow cytometry to measure the effect of metformin on causing cervical cancer cell cycle arrest. As shown in Fig. 1e, the percentage of G0/G1 phase cells increased and the percentage of S phase cells decreased when HeLa and SiHa cells were treated with 20 mM metform for 48 h.

P53 protein is an important regulator of cell proliferation and inhibits HPV E6 protein-induced ubiquitination of proteasome to exert the antitumor effect [26, 27]. Discoidin domain receptor 1 (DDR1) is a collagen binding receptor and also act as an activator of p53 in cancer cell proliferation processes [28]. In this study, SiHa and HeLa cells were treated with varying concentrations of metformin for 48 h, followed by western blotting to assess DDR-1 and p53 expression. As metformin concentration increased from 5 mM to 20 mM, the expression of DDR-1 and p53 protein increased in the cervical cancer cell lines. Metformin upregulated DDR-1 expression activating p53 in the tumor cells in a dose-dependent manner (Fig. 1f). These results suggest that metformin increased DDR-1 expression along with p53 activated to inhibit the proliferation of cervical cancer cells. Taken together, these results indicate that metformin decreased cervical cancer cell proliferation.

### Metformin Upregulates MICA and HSP70 expression to increase the sensitivity of tumor cells to NK cell cytotoxicity

Tumor immune escape is an important feature of tumor invasion and metastasis [25]. Restoring the sensitivity of the body to tumor immune escape is the main means to cancer immunotherapy. MICA is a membrane protein that is involved in anti-infective immunity and antitumor immunity. MICA expression on the tumor cell surface is usually low [29]. When MICA protein is activated on the tumor cell surface, MICA binds to its ligand, NKG2D, on the surface of NK cells to enhance the killing effect of NK cells on tumors [30, 31]. Furthermore, all the other NKG2D ligands, MICB and ULBPs (1 to 6), are also binds to NKG2D to show their antitumor immunity [32].

**Fig. 2:**
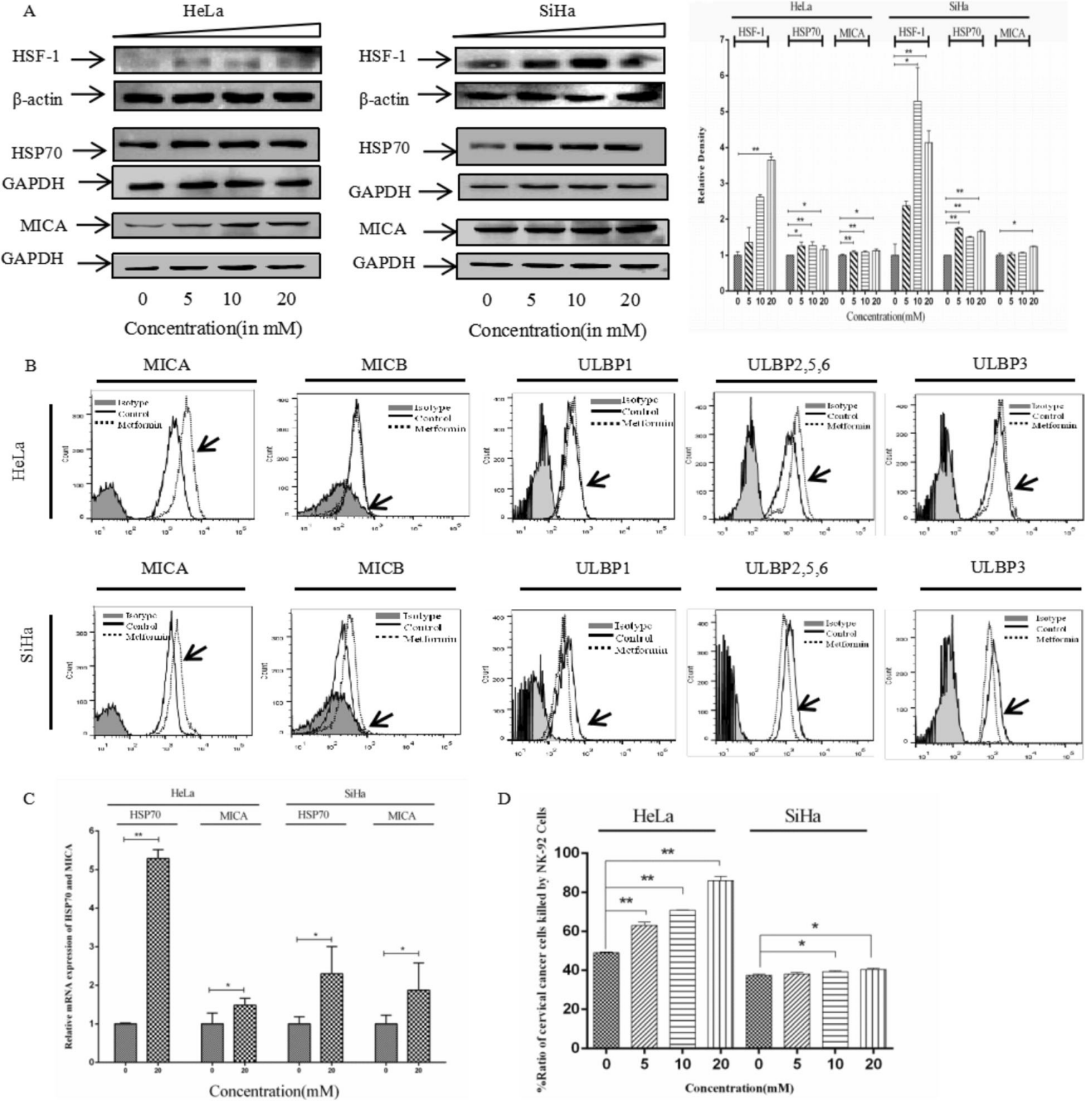
Effect of metformin on MICA and HSP70 protein expression. **a** Representative western blots and a bar chart showing HSF-1, MICA and HSP70 expression in SiHa and HeLa cell after 48 h treatment with metformin of different concentrations; **b** Representative flow cytometry showing MICA, MICB, ULBP1, ULBP2/5/6 and ULBP3 expression in the surface of SiHa and HeLa cell after 48 h treatment with 20 mM metformin. **c** Representative qRT-PCR showing MICA and HSP70 mRNA expression in SiHa and HeLa cell after 48 h treatment with 20 mM metformin. **d** Bar chart showing the enhanced killing rate of NK cells by metformin treatments. Data are presented as mean ± standard deviation, *n* = 3. *P < 0.05, and **P < 0.01, comparison with the 0 μM metformin treatment group

HSP70 is a common molecular chaperone that is expressed in the membranous organelles of all cells. It is mainly involved in protein synthesis and processing [33–35]. Under normal circumstances, HSP70 expression is relatively low; however, under the conditions of protein damage caused by heat shock and hypoxia, HSP70 protein may be regulated by the PI3K/AKT/NRF2 pathway, and its expression is increased [36, 37]. A previous study has shown that HSP70 plays a synergistic role with immune cells in antitumor immunity [38]. Moreover, heat shock factor 1 (HSF-1), one of the best known activators of HSP70 expression, is also an activator of the MICA gene promoter [39]. Therefore, we assessed HSF-1, HSP70 and MICA expression in cervical cancer cells after metformin treatment and also investigated the sensitivity of NK-92 cells to cancer cells. We also evaluated all the other NKG2D ligands expression in the surface of cervical cancer cells. As shown in Fig. 2a and b, metformin increased HSF-1, MICA and HSP70 protein expression in SiHa and HeLa cells and also induced MICA expression on the surface of human cervical cancer cells. However, metformin did not induce MICB, ULBP1, ULBP2/5/6 and ULPB3 expression on the surface of human cervical cancer cells (Fig. 2b). In addition, the mRNA expression of *HSP70* gene and *MICA* gene increased in SiHa and HeLa cells when treated with metformin (Fig. 2c).

We further assessed LDH efflux in the SiHa and HeLa cells and evaluated the effect of metformin on NK cell-mediated killing of human cervical cancer cells. Varying concentrations of metformin treatment (5 mM, 10 mM, and 20 mM) increased the NK-92 cell lethality to SiHa and HeLa cells from 37.45 ± 0.64% to 40.45 ± 0.71(SiHa) and from 49.04 ± 0.32% to 86.00 ± 2.24% (HeLa), respectively, in a dose-dependent manner (Fig. 2d, Table 2). These results suggest that metformin induces MICA and HSP70 expression on the surface of human cervical cancer cells. Additionally, an increase in MICA expression enhanced NK cell cytotoxicity.

**Table 2:**
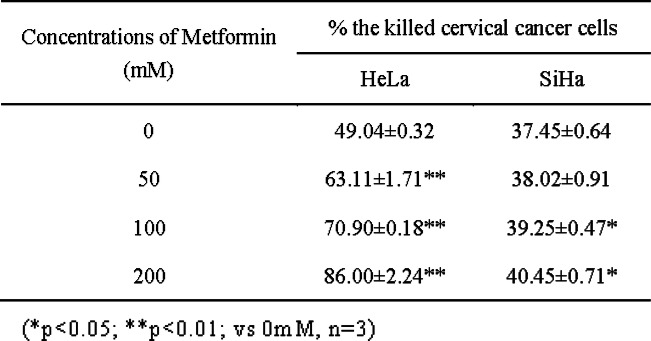
Ration of cervical cancer cells killed by NK-92 cells with different concentrations of metformin

### Metformin increases MICA expression via the PI3K/Akt pathway

**Fig. 3:**
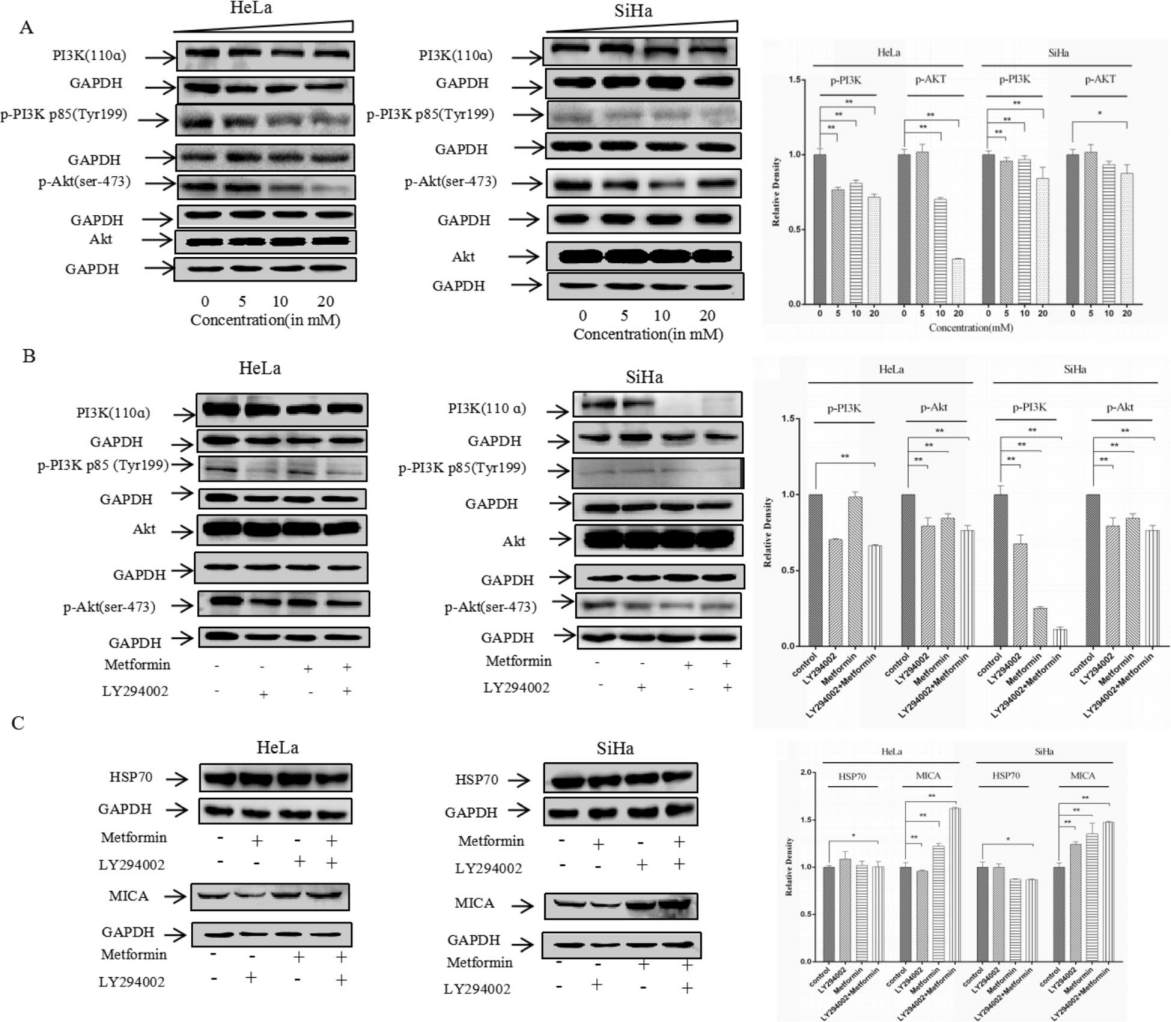
Metformin influences the expression of PI3K (p110ɑ), phospho-PI3Kp55 (Tyr199), and phospho-Akt (ser473) in the PI3K/Akt pathway. **a** PI3K (p110ɑ), phospho-PI3Kp55 (Tyr199), and phospho-Akt (ser473) levels in SiHa and HeLa cell after 48 h treatment with metformin of different concentrations assessed by western blotting; **b** PI3K (p110ɑ), phospho-PI3Kp55 (Tyr199), and phospho-Akt (ser473) levels in SiHa and HeLa cells treated with and without metformin and LY29400 as measured by western blotting; **c** MICA and HSP70 expression in SiHa and HeLa cells treated with and without metformin and LY29400 as measured by western blotting. The data are presented as mean ± standard deviation, *n* = 3. *P < 0.05, and **P < 0.01, comparison with the 0 μM metformin treatment group

Studies have shown that metformin inhibits the invasion and metastasis of tumor cells and induces degradation of cyclin D1 through AMP-activated protein kinase/glycogen synthase kinase 3 beta (AMPK/GSK3β) signaling axis to participate in the protein ubiquitination process [40]. To further elucidate the molecular mechanism by which metformin inhibits the proliferation of cervical cancer cells, SiHa and HeLa cells were treated with varying concentrations of metformin (0 mM, 5 mM, 10 mM, and 20 mM) for 48 h, followed by western blotting to detect PI3K (p110), p-PI3K p85 (Tyr199), Akt, and p-Akt (ser473) protein expression. As the concentration of metformin increased, the expression of p-PI3K p85 (Tyr199) and p-Akt (ser473) decreased (Fig. 3a). When PI3K/Akt signaling was blocked with the PI3K/Akt signaling inhibitor, LY294002, the inhibitory effect of metformin on the p-PI3K p85 (Tyr199) and p-Akt (ser473) was enhanced (Fig. 3b). In addition, after using the LY294002 to block the PI3K/Akt signaling, the induction of MICA by metformin also increased, whereas LY294002 had little effect on HSP70 expression (Fig. 3c). Because metformin targets AMPK signaling and the target of LY294002 is PI3Kɑ/δ/β [41], these different pathways may act synergistically, whereas compounds with the same targets may have antagonistic effects. These results indicate that metformin inhibits tumor cell proliferation by increasing the expression of MICA protein on the tumor cell surface via disrupting the PI3K/Akt pathway.

### Metformin inhibits the growth of cervical Cancer Xenograft in a nude mouse model

**Fig. 4:**
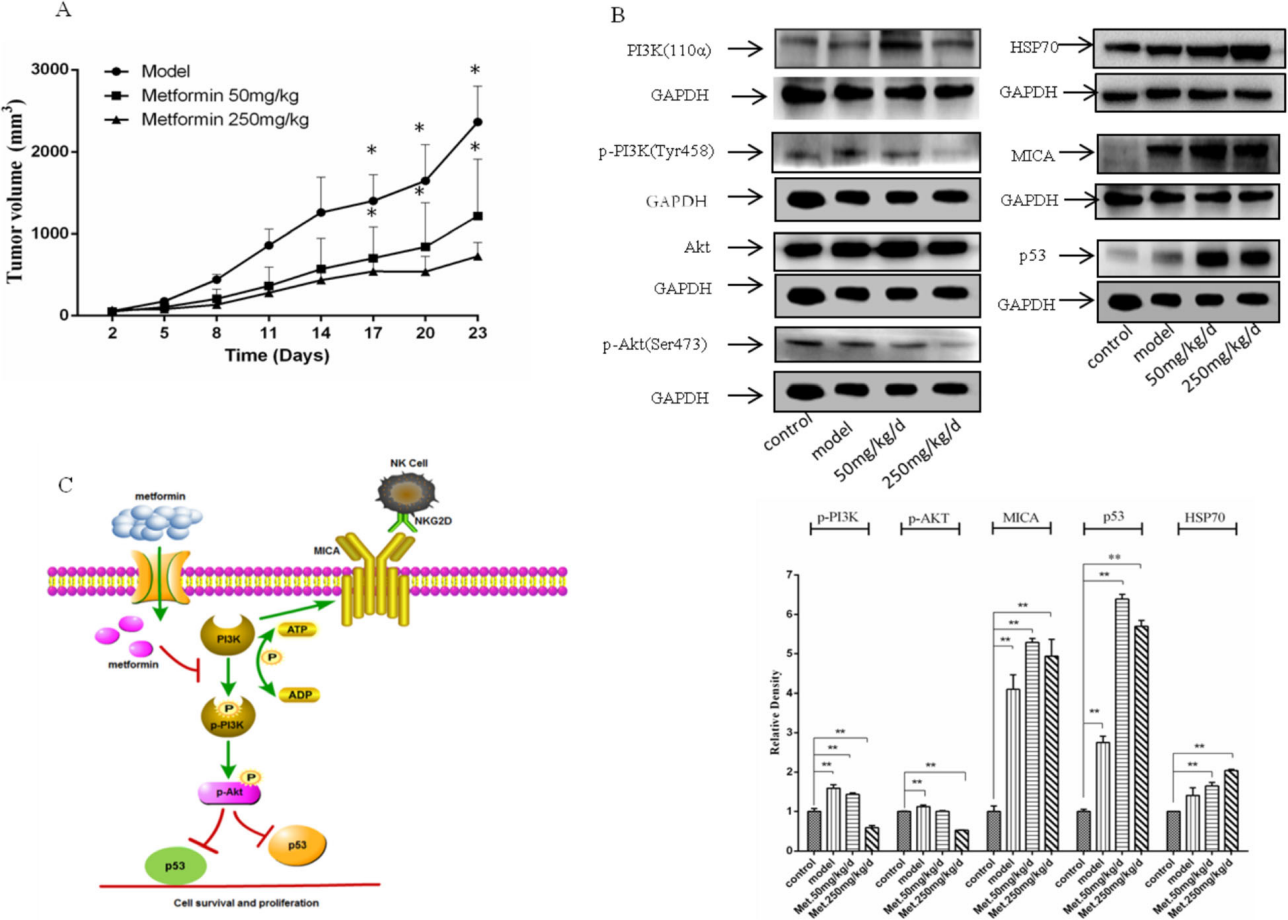
Metformin inhibits the growth of SiHa cell-derived cervical cancer xenografts in a nude mouse model. **a** Inhibition of tumor growth by metformin; **b** Western blotting showing upregulation of MICA and p53 protein expression and inhibition of PI3K (p110ɑ), phospho-PI3Kp55 (Tyr199), and phospho-Akt (ser473) protein expression in xenograft tissues by metformin; **c** Schematic diagram showing the proposed mechanism of metformin, which upregulates MICA expression through the PI3K/Akt pathway and enhances the killing effects of NK cells on tumor cells, thereby improving the recognition of cervical cancer cells by NK cells and disrupting tumor immune escape. In addition, metformin activates p53 expression through the PI3K/Akt pathway to inhibit tumor cell proliferation. The data are presented as mean ± standard deviation, *n* = 3. *P < 0.05, and **P < 0.01, compared to the 0 μM metformin treatment group

Tumor cell line-derived xenograft animal modeling has been extensively used in antitumor drug screening and evaluation because such models are easy to construct, the tumor formation rate is high, and the experimental cycle is short [8]. In this study, SiHa cells were used to develop a cervical cancer xenograft model in BALB/c nude mice to study the inhibitory effect of metformin on tumor growth. After successful modeling, 50 mg/kg/d and 250 mg/kg/d metformin were administered (by oral gavage) to mice bearing cervical cancer xenografts for 23 consecutive days (Fig. 4). Compared to the model group, the mice in the 250 mg/kg/d metformin treatment group exhibited slower tumor growth. Assessment of PI3K (p110), p-PI3K p85 (Tyr199), Akt, p-Akt (ser473), MICA, HSP70 and p53 expression in the tumor xenografts showed that xenografts of the 250 mg/kg/d metformin treatment group had significantly lower p-PI3K p85 (Tyr199) and p-Akt (ser473) levels, whereas the MICA, HSP70, and p53 expression in the xenografts of the 250 mg/kg/d metformin treatment group was significantly higher, compared to the model group. These results suggest that metformin inhibits p-PI3K p85 (Tyr199) and p-Akt (ser473) expression; upregulates MICA and p53 expression; and inhibits the growth of cervical cancer xenografts in mice.

## Discussion

Classic drug design strategies generally target a single protein or signaling pathway. However, the pathogenesis of most diseases, including cancers, is complex. Thus, it is necessary to develop drugs that target multiple proteins and disease-related signaling pathways. Metformin, a biguanide blood sugar-reducing agent, has been extensively used in clinical practice because of its safety and efficacy profile, as well as being inexpensive. Studies have shown that metformin can target one or more signaling pathways to inhibit tumor cell proliferation, invasion, and migration [42]. For example, metformin directly activates the AMPK pathway and reduces insulin-like growth factor 1 (IGF-1) expression to inhibit insulin signaling, block the glucose metabolism pathway of tumor, and exert antitumor effect [43, 44]). A recent study identified AMPK O-GlcNAcylation as anti-proliferative mechanism of metformin in cervical cancer [45]. Since HeLa cells do not express LKB1 (which phosphorylates AMPK) [46], it is shown in some studies that this cell line might be resistant to metformin [47]. However, metformin exhibits a complex anti-tumor mechanism for its small molecular weight (MW: 165.6 )[48, 49]). In this study, we found that metformin inhibits cervical cancer cell proliferation in a dose-dependent manner through non-AMPK signaling pathways.

P53 is a tumor suppressor. More than 50% of all malignant tumors have *p53* mutation. The protein encoded by *p53* gene is a transcriptional factor called TP53 that controls the initiation of cell cycle. P53 protein is mainly distributed in the nucleoplasm of cells and specifically binds to DNA. Its activity is regulated by post-translational modification, such as phosphorylation, acetylation, methylation, and ubiquitination [26, 50]. Normal p53 acts as the “guardian of the genome,” screening for sites of DNA damage in the G1 phase and monitoring the integrity of the genome. In the event of DNA damage, p53 prevents DNA replication to provide sufficient time for the repair of the damaged DNA. It triggers apoptosis if the repair of the damaged DNA fails. During the cell cycle, p53 mainly functions in the monitoring of the G1 and G2/M phase check points, and this function is closely related to transcriptional activation. Discoidin domain receptor 1 (DDR1) can activate p53 by binding its receptor. In our present study, we showed the evidence that metformin upregulated DDR-1 expression inhibiting the proliferation of cervical cancer cells, promoting cervical cancer apoptosis and suppress cervical cancer xenograft tumor tissues growth in a dose-dependent manner along with activating p53.

MICA is a member of the MHC class I molecular family and is expressed on the cell surface membranes. Recent research has shown that MICA is associated with the development of a variety of tumors. It is a stress marker and is expressed in pathogenic bacteria, tumors, and organ transplant recipients [30]. MICA is the receptor of NKG2D, an important activating protein on the NK cell surface, and NK cells play a very important role in tumor innate immunity to kill tumor cells by recognizing tumor cell surface markers and producing a cytotoxic effect [51]. Previous studies have shown that high glucose protects pancreatic cancer from NK cell-mediated killing through suppressing MICA/B expression. Moreover, high glucose inhibited AMP-activated protein kinase signaling, leading to high expression of Bmi1, a polycomb group (PcG) protein which was found to be up-regulated by high glucose, and mediated the inhibition of MICA/B expression through promoting GATA2 in pancreatic cancer [52]. AMP-activated protein kinase (AMPK)–histone deacetylase 5 (HDAC5) pathway promoted nuclear accumulation of HIF-1a and functional activation of HIF-1 by deacetylating heat shock protein 70(HSP70) in the cytosol, indicating a novel link between AMPK, HIF-1a, and HSP70 [53]. HSP70 is not immunogenic but can participate in antigen processing after binding to polypeptides and enhance the immune response. A clinical trial injected purified HSP70 into children with brain cancer and found an antitumor effect and certain feasibility and safety in the antitumor therapy [54, 55]. Another study has also shown that HSP70 inhibits brain tumor development in a C6 glioblastoma mouse model, which is associated with NK cell and T lymphocyte killing abilities of glioblastoma, as well as increased activities of NK cells and CD81^+^ T lymphocytes [55]. In the present study, we found that metformin increased HSF-1, MICA and HSP70 protein expression in SiHa and HeLa cells and also induced MICA expression on the surface of human cervical cancer cells. However, metformin did not induce MICB, ULBP1, ULBP2/5/6 and ULPB3 expression on the surface of human cervical cancer cells. In addition, the mRNA expression of *HSP70* gene and *MICA* gene increased in SiHa and HeLa cells when treated with metformin. We concluded that metformin activated NK cells by regulating MICA though a transcription mechanism to mediate innate immunity. As an immunopotentiator, merformin could upregulate HSF-1, one of the best known activators of HSP70 expression, to activate HSP70 and is involved in specific immune activation in the nude mice and exerted antitumor effect.

In the classic PI3K/Akt/mTOR pathway, PI3K is a family of many lipid kinases consisting of a regulatory subunit, p85, and a catalytic subunit, p110. When a ligand binds to the membrane receptor, the receptor activates p53 and recruits p110, thereby catalyzing the formation of phosphatidylinositol 3-phosphate (PI3P) by phosphatidylinositol 4, 5-bisphosphate (PIP2) on the inner surface of the membrane. PI3P, as a second messenger, further activates Akt and phosphoinositide-dependent kinase 1 (PDK1). Akt, also known as protein kinase B (PKB), is an important downstream molecule of PI3K. Akt immediately attained its activated state by phosphorylation (p-Akt) and activated Akt and mTOR are indicators of poor prognosis in cervical cancer patients. They play an important role in regulating cell growth, proliferation, survival, and glucose metabolism [56, 57]. A previous study has shown that PI3K is highly expressed in a variety of tumors, including cervical cancer [56]. When PI3K is inhibited, the corresponding expression of downstream Akt and mTOR is reduced, and the cell signaling pathway is blocked. Therefore, cell proliferation is inhibited. Although Akt is the core molecule of this signaling pathway, only few small-molecule inhibitors directly inhibit Akt by suppressing its phosphorylation. In the present study, we did not block mAbs for NKG2D and /or MICA to demonstrate the specific activity on the NKG2D receptor. However, we used LY294002, a specific PI3K inhibitor, to block the PI3K/Akt pathway. The results showed that combining metformin with LY294002 showed synergistic effect on upregulating MICA in cervical cancer cells. In general, combining two active molecules having the same target in a signal pathway may result in antagonistic effect, while combination of two active molecules having different targets may lead to synergistic effect [58]. That is to say, metformin directly reduced p-PI3K p85 (Tyr199) and p-Akt (ser473) levels in cervical cancer cells, which probably resulted in the antitumor effect. According with our previous study [59], metformin did not target PI3Ks or Akt.

According to the clinical application guidelines for metformin [60], the daily dose of metformin should not exceed 2000 mg/d. Based on the Meeh-Rubner conversion formula and mouse and human bodyweights and specific surface areas as well as previous studies on tumor growth inhibition by metformin, we find the appropriate doses of metformin intraperitoneal injection to be 50–250 mg/kg/d. We selected low and high doses of metformin, 50 mg/k/d and 250 mg/kg/d, respectively, which were equivalent to 384.5 mg/d and 1922.5 mg/d in human, with the high dose nearly matching the highest dose of metformin in clinical practice. This study showed that metformin significantly reduced the tumor xenograft size in the treatment groups, compared with the model group, suggesting that metformin inhibited PI3K/Akt signaling, upregulated p53 signaling, activated MICA protein expression in the tumor cells and inhibited the growth of cervical cancer xenografts in the BALB/c nude mouse model.

## Conclusions

In short, this study indicated that metformin targets both the PI3K/Akt and p53 pathways and exerts antitumor effects in the body. This study provides insights into the development of multi-target inhibitors for cervical cancer.

## Data Availability

Not applicable.

## Abbreviations

LDH: Lactate dehydrogenase
NK cell: Natural killer cell
MICA: Histocompatibility complex class I-related chain A
PI3K: Phosphatidylinositol 3-kinase
mTORC1: Rapamycin complex 1
Bcl-1: B-cell lymphoma 2
VPA: Valproic acid
HMGA2: High-mobility group A protein 2
